# Four‐Dimensional Computed Tomography Differentiates Congenital Right Pulmonary Vein Atresia From Suspected Arteriovenous Malformation: A Case Report

**DOI:** 10.1002/rcr2.70514

**Published:** 2026-03-01

**Authors:** Takahiro Arano, Shotaro Kanehiro, Hajime Kasai, Toshihiko Sugiura, Hiroki Imabayashi, Yuki Sata, Masayuki Ota, Hidemi Suzuki, Jun‐ichiro Ikeda, Takuji Suzuki

**Affiliations:** ^1^ Department of Respirology, Graduate School of Medicine Chiba University Chiba Japan; ^2^ Department of Medicine, School of Medicine Chiba University Chiba Japan; ^3^ Health Professional Development Center Chiba University Hospital Chiba Japan; ^4^ Department of General Thoracic Surgery, Graduate School of Medicine Chiba University Chiba Japan; ^5^ Department of Diagnostic Pathology, Graduate School of Medicine Chiba University Chiba Japan

**Keywords:** congenital pulmonary vein atresia, four‐dimensional computed tomography, pulmonary vein varix

## Abstract

Congenital pulmonary vein atresia (PVA) is a rare condition often associated with vascular anomalies and complex pulmonary hemodynamics. A 54‐year‐old woman was referred for evaluation of a nodular shadow in the right upper lobe, initially suspected to represent a pulmonary arteriovenous malformation (PAVM). Four‐dimensional enhanced computed tomography (4D‐CT) revealed no abnormal vessels, suggesting a PAVM in the pulmonary arterial phase. However, in the venous phase, the pulmonary vein of the right upper lobe was occluded at the trunk, with reflux via a pulmonary vein varix and an abnormal vein draining into the pulmonary vein of the right middle lobe. Due to the risk of pulmonary hypertension, thromboembolism, or varix rupture, the patient underwent right upper lobectomy. 4D‐CT effectively delineates the vascular morphology by separating the pulmonary arterial and venous phases. Congenital PVA may involve pulmonary vein varices and abnormal vascular formation; 4D‐CT may be valuable for diagnosis and treatment planning.

## Graphical abstract

Congenital pulmonary vein atresia (PVA) is a rare condition often associated with vascular anomalies and complex pulmonary hemodynamics. We present a case in which 4D‐CT effectively differentiated congenital right PVA, presenting with a varix and abnormal vein, from a suspected pulmonary arteriovenous malformation.

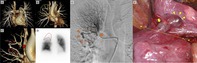

## 1 Introduction

Chest computed tomography (CT) occasionally reveals lung lesions initially suspected to be pulmonary arteriovenous malformations (PAVMs), which, upon detailed evaluation, prove to be other vascular anomalies [1]. Accurate characterisation of pulmonary artery and vein involvement is crucial when lung vascular malformations, including PAVMs, are suspected. Pulmonary vein atresia (PVA) and pulmonary varices can mimic PAVMs, often posing diagnostic challenges with standard contrast‐enhanced CT [2]. However, four‐dimensional computed tomography (4D‐CT) can effectively separate pulmonary arterial and venous phases, even in cases with complex vascular morphology.

We present a case in which 4D‐CT effectively differentiated congenital right PVA, presenting with a varix and abnormal vein, from a suspected PAVM.

## 2 Case Report

**FIGURE 1:**
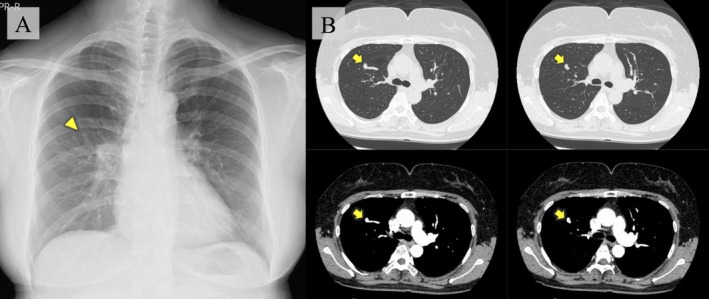
(A) Chest radiograph showing a small nodule in the right middle lung field with well‐defined margins in continuity with the blood vessels (triangle). (B) Contrast‐enhanced chest computed tomography showing an abnormal vascular picture, suspicious for pulmonary arteriovenous malformation (arrow).

A 54‐year‐old woman visited a nearby hospital after an abnormal lung shadow was detected during a routine health check. Chest radiography revealed a nodular opacity in the right upper lung field (Figure 1A).

**FIGURE 2:**
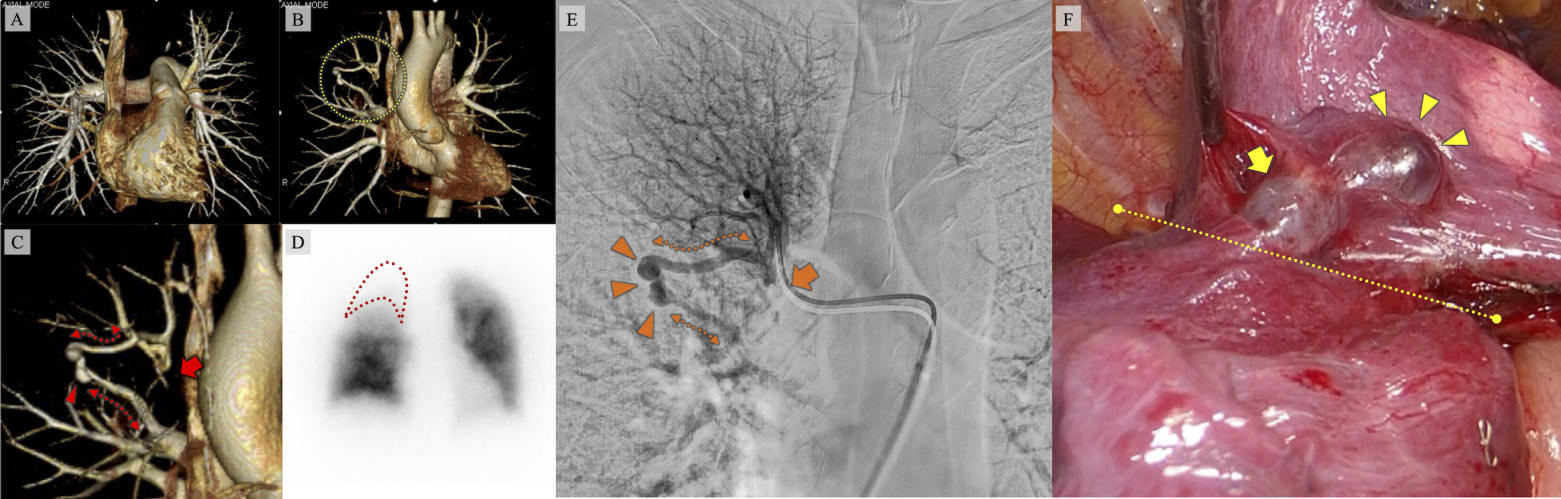
(A) No abnormal vessels detected in the pulmonary arterial phase of the four‐dimensional reconstructed enhanced chest computed tomography (4D‐CT). (B) In the pulmonary venous phase of 4D‐CT, abnormal vessels are visualised in the right upper lobe (dotted area). (C) In the same phase, occlusion of the right pulmonary vein (arrow) is seen, with an abnormal pulmonary vein branching upstream of the occluded segment (dotted line) and rejoining the pulmonary vein in the middle lobe via a pulmonary vein varix (triangle). (D) Pulmonary perfusion scintigraphy showing no abnormal hyperaccumulation in organs outside the lungs, with decreased blood flow in the right upper lobe (dotted area). (E) Selective pulmonary arteriography of the right upper lobe revealing right pulmonary vein occlusion (arrow) with pulmonary venous varix (triangle) and abnormal vessels (dotted line). (F) Intraoperative view showing the pulmonary venous varix along the interlobar fissure (triangles) and a cross‐fissural venous channel (arrow); the interlobar fissure is indicated by the dotted line.

She was referred to our hospital for further evaluation. Her medical history included road traffic trauma at the age of 2 years. She was asymptomatic, with oxygen saturation of 98% on room air. Blood tests and arterial blood gas analyses in ambient air were unremarkable. Contrast‐enhanced chest CT revealed an abnormal nodule associated with the pulmonary vasculature in the right upper lobe (RUL), suggestive of PAVM (Figure 1B). A 4D‐CT scan of the chest showed no abnormalities in the pulmonary artery during the arterial phase (Figure 2A). However, in the venous phase, the pulmonary vein in the RUL was occluded. The abnormal vein drained via the pulmonary vein varix into the pulmonary vein in the middle lobe (Figure 2B,C). Lung perfusion scintigraphy revealed a shunt fraction of 8.0% and hypoperfusion of the RUL (Figure 2D).

Right heart catheterisation demonstrated a mean pulmonary artery pressure at the upper limit of normal (20 mmHg), with pulmonary arterial wedge pressure (11 mmHg), pulmonary vascular resistance (1.8 Wood units), and cardiac index (3.26 L/min/m^2^ on room air), all within normal ranges. Selective pulmonary arteriography of the RUL revealed an occluded pulmonary vein draining into the middle lobe vein via an abnormal vessel and varix (Figure 2E). A diagnosis of pulmonary vein occlusion with abnormal pulmonary vein and varix was made. Right upper lobectomy was performed to prevent pulmonary hypertension due to venous obstruction, thromboembolism, or varix rupture. Intraoperatively, a pulmonary venous varix was identified on the interlobar surface between the right upper and middle lobes, with a venous channel crossing the fissure towards the middle lobe (Figure 2F). As the varix was exposed within the fissure and rupture risk was considered low, right upper lobectomy was performed in a standard sequence. The cause of the occlusion could not be detected from the resected specimen, and congenital PVA was diagnosed.

**FIGURE 3:**
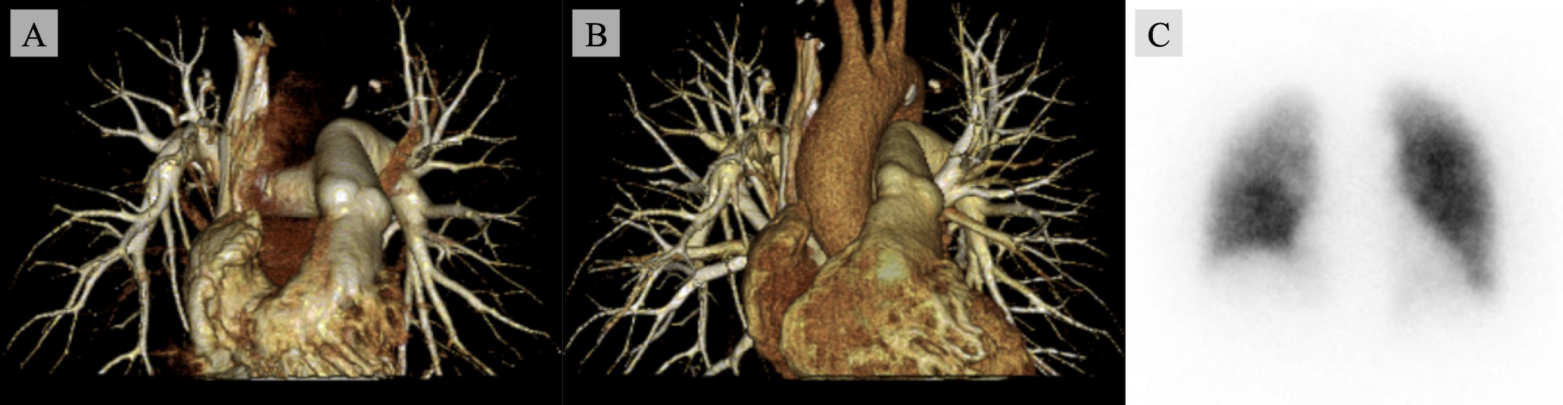
(A) Four‐dimensional reconstructed enhanced chest computed tomography (4D‐CT) at 12 months after surgery shows no abnormal vessels in the pulmonary arterial phase. (B) In the pulmonary venous phase of 4D‐CT, no abnormal vessels are detected. (C) Pulmonary blood flow scintigraphy at 12 months after surgery shows no perfusion defects.

The postoperative course was uneventful, with no respiratory symptoms. At 12 months, follow‐up 4D‐CT showed no recurrence or progression of abnormal vessels (Figure 3A,B), and perfusion scintigraphy demonstrated normal findings (Figure 3C).

## 3 Discussion

This case report highlights two notable clinical findings. First, congenital PVA may coexist with other vascular anomalies, and the risk of potential complications should be considered even in asymptomatic patients. Second, advanced imaging modalities such as 4D‐CT are valuable in evaluating lesions suspected to be PAVMs.

Congenital PVA is rare and may present with associated vascular malformations, though it can remain clinically silent [3]. It is classified into three types according to the pulmonary vein lesion location: common, unilateral, and individual. This case was classified as individual [3]. Individual PVA involves atresia of lobar or segmental pulmonary veins, usually with minimal symptoms [3]. In this case, atresia and abnormal vessels developed as collateral blood vessels, one forming a pulmonary vein varix. Inadequate venous return through these collaterals may have resulted in a mild elevation in pulmonary arterial pressure. Pulmonary varices are typically asymptomatic [4]. Despite the coexistence of PVA, abnormal pulmonary vein, and varix, this patient remained asymptomatic. However, reports exist regarding complications arising from the varix and an abnormal pulmonary vein, such as haemoptysis, middle lobe syndrome due to bronchial compression, dysphagia resulting from oesophageal compression, and cerebral infarction due to thrombus in the varices [5]. Given these risks, the patient underwent a right upper lobectomy.

4D‐CT is a valuable tool for assessing vascular abnormalities, even in cases where multiple pulmonary vascular anomalies coexist. In approximately 10% of cases with suspected PAVMs on chest CT, pulmonary angiography failed to demonstrate a malformation between the pulmonary artery and pulmonary vein [1]. Pulmonary venous abnormalities are especially prone to misdiagnosis as PAVMs [1]. Mimickers of PAVMs on chest CT include arterial and venous lesions, both intrapulmonary and extrapulmonary [2]. These include extrapulmonary arteriovenous malformations, pulmonary vein abnormalities, occluded or stenotic pulmonary veins, partial anomalous pulmonary venous return, pulmonary varices, and pulmonary aneurysms [2]. Therefore, if a PAVM is suspected, 4D‐CT and pulmonary angiography can be useful for evaluating the pulmonary arteries and veins. Moreover, 4D‐CT provides both arterial and venous phase imaging with minimal invasiveness. Distinguishing PVA from PAVM is clinically important, as management strategies differ. Key imaging features and management implications are summarised in Table 1. In this case, a PAVM was initially suspected based on contrast‐enhanced CT findings. However, detailed morphological evaluation with 4D‐CT confirmed the diagnosis of pulmonary vein occlusion, with an abnormal pulmonary vein and varix.

**TABLE 1:** Key imaging features and management implications for suspected cases of PAVM and common mimickers.

Entity	Imaging findings			Management
	Arterial phase	Venous phase	Morphology	
PAVM	Homogeneously enhancing dilated vascular structure, isoattenuating to the feeding and draining vessels.	Pulmonary veins in segments or lobes other than the AVM‐bearing region become opacified.	Well‐defined peripheral nodule with a feeding pulmonary artery and one or more draining pulmonary veins.	Transcatheter embolization.
PVA with collateral vein(s)^a^	Not specific.	Venous drainage detours via collaterals to a normal pulmonary vein/left atrium; collaterals may be fine or dilated/tortuous.	Atresia or stenosis of one or more pulmonary veins; the individual type involves lobar or segmental pulmonary veins.	Surgery or balloon angioplasty if symptomatic.
Pulmonary vein varix^a^	Not specific.	The anomalous vein fills at the same rate as normal pulmonary veins, but drainage to the left atrium may be delayed compared with normal veins.	Aneurysmal dilatation of a pulmonary vein; typically an anomalously dilated perihilar vein with sparing of the periphery.	Annual monitoring for possible complications.
PAPVR	Not specific.	The anomalous drainage pathway and systemic venous or right atrial connection are identified.	One or more (but not all) pulmonary veins drain into the systemic venous system or the right atrium.	Surgery if a significant left‐to‐right shunt is present.
Pulmonary artery aneurysm	Focal enhancing aneurysmal dilatation contiguous with the pulmonary artery (main pulmonary artery and/or branches).	No early opacification of the draining vein, unlike PAVM.	Focal aneurysmal dilatation of the main pulmonary artery and/or branches.	Surgery or endovascular treatment if enlarging or symptomatic.
Extrapulmonary AVM (pleural or mediastinal AVM)	Peripherally located enhancing vascular tangle abutting the pleural surface.	Drains mainly into systemic veins (e.g., intercostal, subclavian, or innominate veins).	Extrapulmonary nidus located in pleural or mediastinal spaces (high‐flow vascular malformation).	Sclerotherapy of nidus.

Abbreviations: AVM, arteriovenous malformation; PAPVR, partial anomalous pulmonary venous return; PAVM, pulmonary arteriovenous malformation; PVA, pulmonary vein atresia.

^a^ Entity in the present case.

In conclusion, congenital PVA may be associated with multiple vascular malformations, which can be challenging to differentiate from PAVMs on contrast‐enhanced CT. Accurate evaluation of vascular morphology requires assessment of both the pulmonary arterial and venous phases using imaging modalities, including 4D‐CT.

## Author Contributions

Dr. Takahiro Arano is the guarantor of this manuscript and contributed to the writing and review of the entire manuscript. Drs. Shotaro Kanehiro, Hajime Kasai, Toshihiko Sugiura, and Takuji Suzuki critically reviewed the manuscript. Drs. Hiroki Imabayashi, Yuki Sata, Masayuki Ota, Hidemi Suzuki and Jun‐ichiro Ikeda collected and analysed the clinical data of the patient and critically reviewed the manuscript. All authors approved the final version of the manuscript.

## Funding

The authors have nothing to report.

## Consent

The authors declare that written informed consent was obtained for the publication of this manuscript and accompanying images and attest that the form used to obtain consent from the patient complies with the Journal requirements as outlined in the author guidelines.

## Conflicts of Interest

The authors declare no conflicts of interest.

## Data Availability

The data that support the findings of this study are available on request from the corresponding author. The data are not publicly available due to privacy or ethical restrictions.
